# Detecting the Information of Functional Connectivity Networks in Normal Aging Using Deep Learning From a Big Data Perspective

**DOI:** 10.3389/fnins.2019.01435

**Published:** 2020-01-17

**Authors:** Xin Wen, Li Dong, Junjie Chen, Jie Xiang, Jie Yang, Hechun Li, Xiaobo Liu, Cheng Luo, Dezhong Yao

**Affiliations:** ^1^College of Information and Computer, Taiyuan University of Technology, Taiyuan, China; ^2^The Clinical Hospital of Chengdu Brain Science Institute, MOE Key Lab for Neuroinformation, University of Electronic Science and Technology of China, Chengdu, China; ^3^School of Life Sciences and Technology, University of Electronic Science and Technology of China, Chengdu, China

**Keywords:** deep learning, big data, DNN, functional connectivity, aging

## Abstract

A resting-state functional connectivity (rsFC)-constructed functional network (FN) derived from functional magnetic resonance imaging (fMRI) data can effectively mine alterations in brain function during aging due to the non-invasive and effective advantages of fMRI. With global health research focusing on aging, several open fMRI datasets have been made available that combine deep learning with big data and are a new, promising trend and open issue for brain information detection in fMRI studies of brain aging. In this study, we proposed a new method based on deep learning from the perspective of big data, named Deep neural network (DNN) with Autoencoder (AE) pretrained Functional connectivity Analysis (DAFA), to deeply mine the important functional connectivity changes in fMRI during brain aging. First, using resting-state fMRI data from 421 subjects from the CamCAN dataset, functional connectivities were calculated using sliding window method, and the complex functional patterns were mined by an AE. Then, to increase the statistical power and reliability of the results, we used an AE-pretrained DNN to relabel the functional connectivities of each subject to classify them as belonging to the attributes of young or old individuals. A method called search-back analysis was performed to find alterations in brain function during aging according to the relabeled functional connectivities. Finally, behavioral data regarding fluid intelligence and response time were used to verify the revealed functional changes. Compared to traditional methods, DAFA revealed additional, important aged-related changes in FC patterns [e.g., FC connections within the default mode (DMN) and the sensorimotor and cingulo-opercular networks, as well as connections between the frontoparietal and cingulo-opercular networks, between the DMN and the frontoparietal/cingulo-opercular/sensorimotor/occipital/cerebellum networks, and between the sensorimotor and frontoparietal/cingulo-opercular networks], which were correlated to behavioral data. These findings demonstrated that the proposed DAFA method was superior to traditional FC-determining methods in discovering changes in brain functional connectivity during aging. In addition, it may be a promising method for exploring important information in other fMRI studies.

## Introduction

Functional networks (FNs) constructed by resting-state functional connectivity (rSFC) analysis using data from blood oxygen level dependence (BOLD)-based functional magnetic resonance imaging (fMRI) have greatly deepened our understanding of the functioning of the human brain. During the resting-state, time series from the same FN are temporally correlated with each other, and the FNs thus reflect functional communication between brain regions. Due to its non-invasiveness and effectiveness, resting-state FC analysis has become the most extensive and important tool for exploring changes in brain function (Biswal et al., 1995; De Luca et al., 2006). With an increasing global emphasis on human health, brain aging has become a research hotspot in brain science. Functional connectivity analysis based on rs-fMRI has been widely applied in brain aging studies (Ferreira and Busatto, 2013). These studies have indicated that the strength of multiple FNs, including the default mode network (DMN) and dorsal attentional network (DAN), decreases during the aging process. (Sheffield et al., 2015; Avelar-Pereira et al., 2017; Vij et al., 2018), the phenomenon of which indicates that attention, memory and executive control functions decline in cognitive aging. Meanwhile, researchers have also found that increased FC involving the frontal, parietal networks and motor, subcortical networks was related to aging, which may reflect the compensatory responses to the decreased strength of FC during aging (Biswal et al., 2010; Tomasi and Volkow, 2012; Ferreira and Busatto, 2013). These results have greatly enhanced our understanding of aging in the brain; functional connectivity analysis is expected to be a valuable tool for objectively evaluating the health status of the brain in the function and cognition of elderly individuals and thus may benefit the clinical diagnosis and intervention of brain aging-related diseases (Geerligs et al., 2017; de Vos et al., 2018). However, most of the present studies primarily rely on traditional, relatively small samples (from 10 to a few 100 samples) based on statistical analyses (e.g., two-sample *t*-test and multiple linear regression), which may result in insufficient analysis of resting-state FCs during brain aging, thus ignoring some important underlying information regarding FN alterations.

Machine learning is a set of algorithms used for the automatic development of models and learning of complex patterns from data. Over the past decade, machine learning has achieved great success in mining functional connectivity. For example, Calhoun et al. performed independent component analysis (ICA) to decompose fMRI data into reduced-dimensional time series and spatial patterns, which were further used for modal fusion and classification prediction (Calhoun and Sui, 2016). Du et al. (2017) proposed a new scheme of group information-oriented ICAs to mine dynamic functional connectivity from the fMRI data of patients with various mental disorders, detecting the differences of brain function between groups related to diseases, which were not found by traditional static methods. However, traditional linear machine learning algorithms rely on feature extraction, which cannot handle non-linear and complex relationships in data, and lack the ability to process FCs directly as well (Plis et al., 2014). Therefore, deep learning methods developed based on machine learning is gradually attracting the attention of neuroscience researchers. Accumulating evidence shows that deep learning is superior to traditional machine learning with regard to fMRI-related pattern recognition (Hu et al., 2018), dimensionality reduction (Suk et al., 2016; Kawahara et al., 2017), classification (Kim et al., 2016; Guo et al., 2017), and prediction (Chae et al., 2018; Khosla et al., 2019). For example, in terms of non-linear pattern recognition, Suk et al. (2016) applied auto-encoders to extract the dynamic changes in brain function from a time series of fMRI. These changes have significant sensitivity in distinguishing individuals with mild cognitive impairment from healthy people. For classification, Kim et al. (2016) used a DNN to mine the resting-state FNs with physiological significance, utilized mining features to classify schizophrenic patients and health controls, and obtained an accuracy of classification that was 22.3% higher than that of a support vector machine (SVM). However, the number of data samples used in the training models of these studies was still relatively small (<100), and so it is possible that these methods missed some important information due to inadequate learning (Xia and He, 2017).

With the continuous deepening of collaborative research on global brain aging, research based on open data sets [e.g., Cambridge Centre for Ageing and Neuroscience (CamCAN) dataset^1^ ] makes it possible to study brain aging from a big data perspective. The increase in open data set samples for analysis likely enhances the effectiveness of statistical analysis and facilitates new research designs (Peter and Jayati, 2018; Smith and Nichols, 2018). However, traditional statistics with big data may lead to possible confounding effects (Smith and Nichols, 2018), thus requiring the development of new data analysis methods in the context of big data. Considering the superiority of deep learning and big data analysis to traditional modeling methods, combining deep learning with neuroimaging big data is expected to deeply explore brain aging information by taking into account the increase in sample size and the complex relationships in the data.

Spontaneous brain activity during resting-state is an apparent variability of interaction between brain regions and may be dominated by traces of activity, which may involve different subregions in a network at different times (Liu and Duyn, 2013; Karahanoglu and Van De Ville, 2015). Furthermore, functional connectivity, usually measured by temporal correlations between brain regions, can reflect these functional interactions and yield details maps of complex neural systems (Biswal et al., 1995, 2010). Therefore, we proposed the hypothesis that most of the functional activities in young adults may reflect the “young FCs” (FCs which reflect intrinsic brain activities in young adults), while those in old adults may largely reflect the “old FCs” (FCs which reflect intrinsic brain activities in old adults). Under this hypothesis, traditional static FC method may miss some important information in comparing functional connectivity differences between young and old groups. Meanwhile, previous studies implied that deep learning methods (especially DNN) were superior to traditional machine learning methods (Kim et al., 2016; Hu et al., 2018) both for classification and mining underlying information in fMRI data. Therefore, a new method based on the above-mentioned assumption is expected to detect the information of functional connectivity networks in normal aging. In this work, using deep learning from a big-data perspective, we proposed a new analysis method, named DNN with AE pretrained Functional connectivity Analysis (DAFA), to deeply mine the important functional connectivity changes in fMRI during brain aging. A sliding window method was used to increase the sample size of FCs for each subject by ∼100 times (∼hundred thousand) in order to meet the requirements of deep learning from a big data perspective to a certain extent. After calculating the resting state FCs of each subject at different time windows, the FCs are first fed to DAFA to train the AE-pretrained DNN model with a Softmax layer as a classifier to relabel all FC samples to “young FCs” or “old FCs.” Then, according to the predicted labels, we compared the percentages of “young FCs” between young and old groups using a two-sample *t*-test and further investigated the differences between the mean “young FCs” in the young group and the mean “old FCs” in the old group.

## Materials and Methods

### Participants

In this study, as shown in Table 1, fMRI data was obtained from a total of 412 individuals (after quality control, e.g., excluding excessive head motion, missing T1 images, etc.), which included 220 young adults whose ages ranged from 18 to 45 and 192 elderly adults whose ages ranged from 66 to 88. The binary young/old classes were based on the published paper of Cam-CAN open dataset (Shafto et al., 2017). In each group, the number of participants was approximately equal when divided into 10 year-wide bins. The neuroimaging data used were a subset of the Cambridge Centre for Ageing and Neuroscience (see text footnote 1) (Taylor et al., 2017). Participants performed cognitive tasks outside the MRI scanner. The tests used in this study were the Cattell Culture Fair test, used to assess fluid intelligence (Horn and Cattell, 1966), and the speed Choice Reaction Time (RT) task, used to assess speed of processing. For the RT tasks, the mean (M-RT) and variability (SD of RT values, SD-RT) were computed from individual trials. Ethical approval for the study was obtained from the Cambridgeshire 2 (now East of England – Cambridge Central) Research Ethics Committee. All participants gave written informed consent.

**TABLE 1:** Participants’ demographic information.

	**Group**	**Number**	**Range**	**Mean**	**SD**
Age^∗^	Young	220	18–45	30.25	5.82
	Old	192	66–88	75.29	6.17
Cattell^∗^	Young	213	22–44	36.59	4.35
	Old	188	11–39	26.08	6.04
M-RT^∗^	Young	215	0.27–0.39	0.31	0.05
	Old	140	0.29–0.48	0.34	0.07
SD-RT^∗^	Young	215	0.07–0.17	0.12	0.02
	Old	140	0.01–0.30	0.08	0.04

*^∗^indicates a significant difference between groups using two-sample *t*-test, *p* < 0.001.*

### fMRI Acquisition and Preprocessing

Imaging data were collected by a 3 T MRI scanner (Siemens TIM Trio). During the scanning period, all participants were required to lie still and keep their eyes closed. The T1-weighted anatomical images were gathered using an MPRAGE sequence with the following parameters: TR/TE = 2250 ms/2.99 ms; flip angle = 9°; FOV = 256 × 240 × 192 mm^3^; voxel size = 1 × 1 × 1 mm^3^. For rs-fMRI measurements, 261 volumes of echo planar imaging (EPI) sequences were acquired with the following parameters: sequential descending order; slice thickness 3.7 mm with a slice gap of 20% for whole-brain coverage; TR/TE = 1970 ms/30 ms; flip angle = 78°; FOV = 192 × 192 mm^2^; voxel size = 3 × 3 × 4.44 mm^3^; number of slices = 32; duration time = 520 s.

To remove T1 saturation effects, the first five volumes were deleted from the resting-state fMRI data of each subject. Then, SPM12^2^ and NIT http://www.neuro.uestc.edu.cn/NIT.html (Dong et al., 2018) were used to preprocess the resting-state fMRI data. The fMRI preprocessing contained the following steps: realignment, slice time correction, spatial normalization using T1-weighted MRI data (3 × 3 × 3 mm^3^) and smoothing [6-mm full-width at half-maximum (FWHM)]. Nuisance noises such as linear trends, 12 head-motion parameters, global signals, and individual mean WM and CSF signals were removed using multiple linear regression analysis, and temporal bandpass filtering (pass band 0.01–0.08 Hz) was conducted on the fMRI data. The head motion of each participant was calculated using the mean framewise displacement (mean FD) (Power et al., 2012). Participants whose FD was two or more SD above the group mean FD were excluded from further analysis.

### Method Overview

**FIGURE 1:**
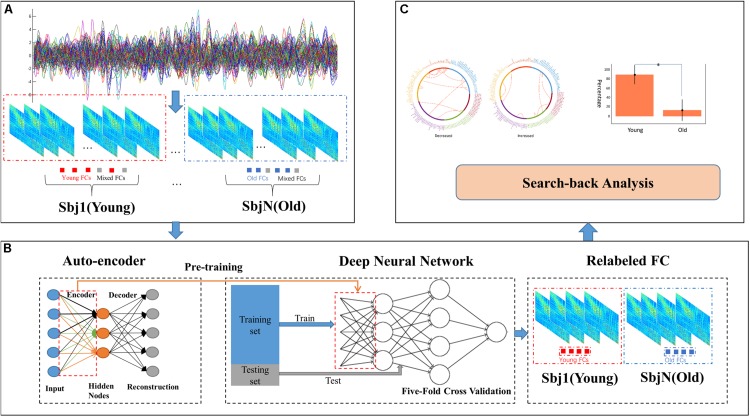
Workflow of the DAFA method. **(A)** Constructing FCs using sliding windows and Pearson’s correlation methods, all the FCs from a subject contains most of “young FCs”/“old FCs” and mix FCs. **(B)** Relabeling all FCs via a DNN based on AE pretraining, and then calculating the average FC of “young FCs” in young subject and “old FCs” in old subject as this subject’s FC. **(C)** Performing a search-back analysis to compare the percentages of “young FCs” and functional alterations between the young and old groups.

In this paper, a method named DAFA was used to locate significant differences in functional connectivity between the old and young groups. Figure 1 shows the workflow of the proposed procedure.

The entire procedure consisted of three steps. (A), whole-brain functional connectivity patterns based on sliding windows (window length: 50 time points, window step: 1 time point) were calculated by the Pearson’s correlation coefficient of the time courses from every pair of 160 ROIs (Dosenbach et al., 2010), followed by Fisher’s r-to-z transformation. (B), an autoencoder was trained on the whole FC dataset using gradient descent learning with L_2_ norm regularization. Then, the weights learned from the AE were set as an initialization of the parameters of the bottom layer to a DNN with a Softmax classifier as the top layer. DNN was used to relabel the FC samples to deeply mine the important functional connectivity changes between young and old groups (which may be not well captured with conventional method), under the condition of mixed labels. FCs in the dataset were labeled as “young FCs” or “old FCs” by the DNN with five-fold cross validation (the cross-validation procedure was performed on subjects and each subject contained 212 FCs via sliding window method, FCs from a subject were either divided to training set or testing set). (C), a search-back analysis procedure was performed to reveal the functional alterations between the mean young FC in the young group and the mean old FC in the old group and the differences between the percentages of “young FCs” labeled in the young group and the old group.

### Autoencoder

The architecture of an autoencoder is a three-layer feed-forward neuro network that contains an input layer, a hidden layer, and an output layer. All three layers are fully connected hierarchically through weighted connections that are updated by a back-propagation algorithm. An important property of the AE’s structure is that the dimensions of the input layer and output layer are the same, while the dimension of the hidden layer is much smaller so that the output layer is forced to reconstruct the pattern of the input layer with the smaller size of the hidden nodes, the value in each hidden node could represent a low-dimension feature from the input data, and the whole AE could be interpreted as a dimension-reduction function.

The basic principle behind training the AE is the minimizing of the residual error between the values of the input and output layers. Let *X* = [*x*_1_,*x*_2_,*x*_3_,…,*x*_*d*_] denote all the FCs for the entire dataset, *y* = [*y*_1_,*y*_2_,*y*_3_,…,*y*_*p*_] denote the features that represent × after dimension reduction, and *z* = [*z*_1_,*z*_2_,*z*_3_,…,*z*_*d*_] denote the corresponding reconstructed data. The number of input and output layer nodes is indicated by d, whose size is equal to the number of observations, and the number of hidden nodes is denoted by *p* (*p* < *d*); the weight and bias of the encoder and decoder can be denoted by *w*^(1,0)^ ∈ *R*^(*p*×*d*)^,*b*^(1,0)^ ∈ *R^p^* and *w*^(2,1)^ ∈ *R*^(*d*×ρ)^, *b*^(2,1)^ ∈ *R^d^*. The features are computed by *y* = *f*(*w*^(1,0)^*x* + *b*^(1,0)^) where *f*(*x*) is a sigmoid function of the hidden nodes; the reconstructed data are computed by *z* = *g*(*W*^(2.1)^*x* + *b*^(2,1)^), where *g*(*x*) is the tanh function of the output nodes. In this paper, we chose autoencoders to perform dimension reduction, and the weights were transferred to the DNN model for pretraining. In every training iteration, a set of edges generated by the Pearson’s correlation of time courses in a window from a sample were fed to the AE, following which the AE was trained using a back-propagation algorithm. After the AE was trained from the whole dataset, the cost function was converged, which ensures that the average reconstruction error through the training set was minimized, resulting in an AE that learned the essential features in a relatively low dimension (from 12700 to 100).

### Relabeling FCs via Training the DNN Model

The DNN model consisted of two hidden layers and a Softmax layer as the output layer. The sliding window-generated FCs of the young group were labeled with [1, 0]^*T*^, and those of the old group were labeled with [0, 1]^*T*^. The cost function of the DNN for the supervised fine-tuning step was defined using the cross-entropy loss function, L_2_ norm regularization term as follows:

$$J\,\left(W\right)=\sum_{i=1}^{N}t^{i}\,\log y^{i}\,W+\left(1-t^{i}\right)\,\log\left(1-y^{i}\right)+\frac{1}{2}\,\lambda^{\left(J-1,J\right)}$$

(1) $$\sum_{j=0}^{l}{||W^{\left(J+1,J\right)}||}^{2}$$

Where *y^i^W* is a vector with elements of the output values from the Softmax layer for subject n in the training set, *t^i^* is the target output value of the window of the *i*^*th*^ subject, λ^(*J−1, J*)^ is the parameter of the L_2_ norm regularization term between the *j*^*th*^and (*j* + 1)^*th*^ layer, N is the total number of samples in the training set, and *l* + 1 is the number of layers, including the input layer. The learning rate was initialized to 0.0015, and the parameter of the L_2_ norm was set to 10^–6^ to prevent overfitting. A total of 50 epochs were used, and the number of hidden nodes in layers was set to [100:50:2]. A momentum term was added to the current weight update term to accelerate the learning procedure. Additionally, weights between the input layer and the first hidden layer of the DNN were initialized as weights obtained by the AE and were frozen during the fine-tuning process (pretrain). In this work, we used five-fold cross validation to prevent overfitting. Finally, the predicted labels from each window for each of the subjects in the dataset were obtained (relabeling).

## Results

### Autoencoder Parameter Settings

**FIGURE 2:**
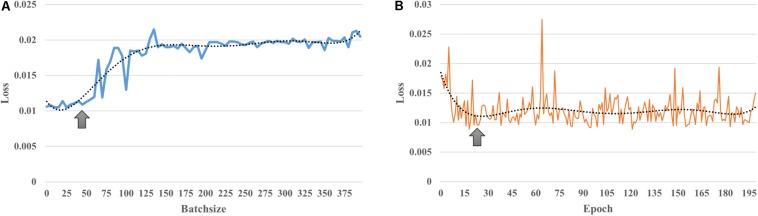
Learnt parameters. **(A)** As the batch size increases, loss increases; **(B)** when the number of epochs = 20, loss reached its first minimum value and then changed periodically with increasing number of epochs.

The input size of our autoencoder was 12720, the number of edges in a subject’s functional connectivity based on 160 ROIs. AE is an unsupervised learning model, and the hyper parameters are essential for the performance in an AE. The purpose of adjusting the value of the parameters is to minimize the reconstructed error. In addition, to avoid identical transformations and enhance the learning ability of the AE, the activation functions used by the encoder and the decoder were the sigmoid function and the tanh function, respectively. As mentioned in the see section “Materials and Methods,” the learning rate and parameter of the L_2_ norm were 0.0005 and 10^–5^, respectively. As shown in Figure 2, the loss increases with a larger batch size; in this study, a batch size of 50 was chosen, as this was the location of the inflection point (Figure 2A); then, the number of epochs was set to 20, corresponding to the first minimum loss value (Figure 2B); in order to minimize the reconstructed error while avoiding redundant features, the number of hidden nodes was empirically set to 100. The initialization of these hyper parameters and learned parameters could best reduce the reconstructed error both in terms of the empirical setup and fine-tuning.

### “Young FCs” in the Young and Old Groups

**FIGURE 3:**
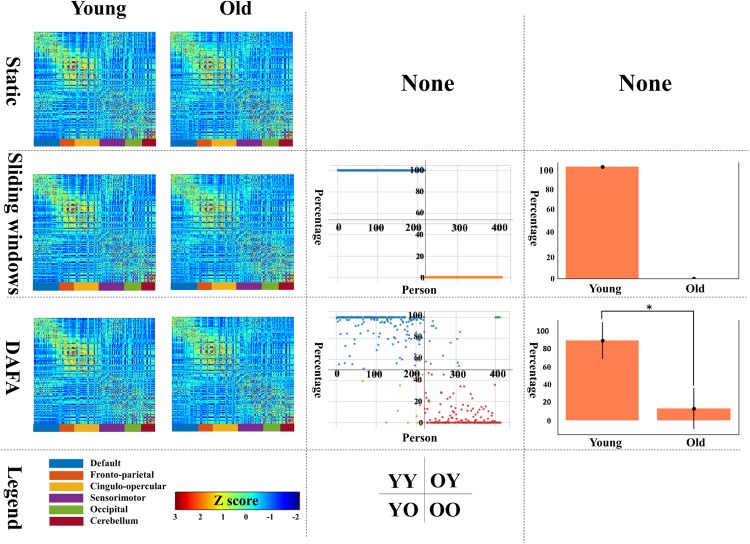
The first column shows the average FC patterns of the young group and old group on the left and right, respectively; the second column shows the distribution of “young FCs” (samples in blue indicate individuals in the young group whose percentage of “young FCs” is greater than 50%; samples in orange indicate individuals in the young group whose percentage of “young FCs” is equal to or below 50%; samples in green indicate individuals in the old group whose percentage of “young FCs” is above 50%; and samples in yellow indicate individuals in the old group whose percentage of “young FCs” is equal to or less than 50%); the third column shows the mean and standard deviation of “young FCs” in the two groups. YY indicates samples in the young group for whom more than 50% of the FCs were relabeled to young; YO indicates samples in the young group for whom more than 50% of the FCs were relabeled to old; OO indicates samples in the old group for whom more than 50% of the FCs were relabeled to old; and OY indicates samples in the old group for whom more than 50% of the FCs were relabeled to young.

The first column of Figure 3 illustrates the average functional connectivity patterns in both the young and old groups using three methods: static, the mean FC of the sliding window and DAFA.

Using the static, sliding window and DAFA methods (the second column of Figure 2), the distributions of “young FCs” were different in both the young and old groups. In the static case, one subject had one static FC, so the percentage of “young FCs” is meaningless. In the sliding window case, all the FCs of an individual subject were classified the same way as that subject’s group (e.g., individuals in the young group only had “young FCs”). When switching to the proposed DAFA, the percentage of “young FCs” in each sample varied on an individual basis.

To evaluate our assumption, the FCs belonging to the young group (“young FCs”) were relabeled using five-fold CV based on each subject. For DAFA, the average percentage of “young FCs” in the young group was 89.8% of the 212 FCs, and the average percentage in the old group was 13.2%. A two-sample *t*-test revealed a significant (*T* = 27.1, *p* = 1.5 × 10^–92^) difference between the percentage of “young FCs” in the young group and in old group after controlling for the covariant variables of gender, head motion parameter (mean FD) and intracranial volume.

### Differences Between “Young and Old FCs”

**FIGURE 4:**
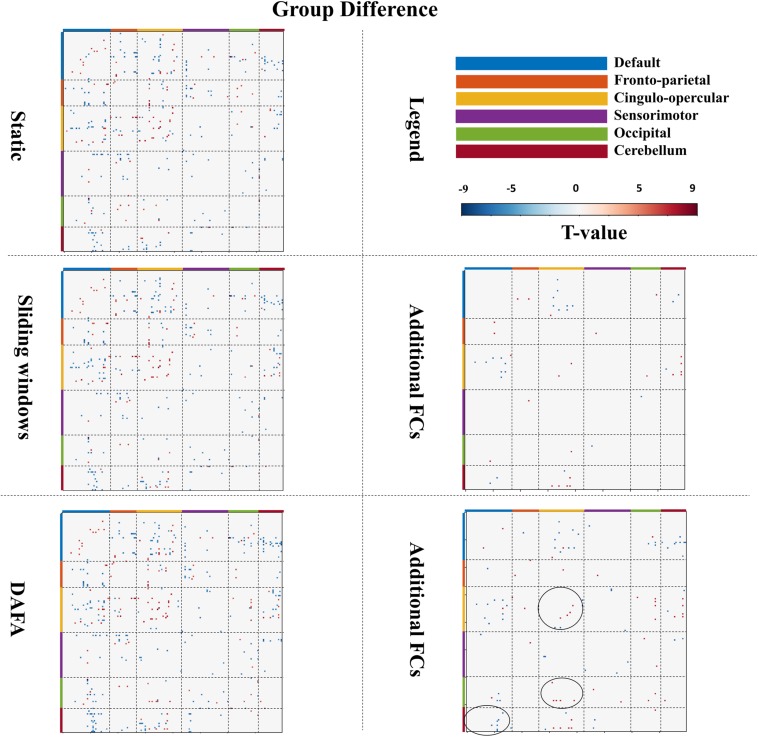
Figures in the **left** column show significant changes in FCs (T-map, *p* < 0.01, FWE corrected) between the young and old groups via the static, sliding windows and DAFA methods. Blue indicates that the FC from the old group is stronger than that from the young group (i.e., old > young), and red indicates that the FC from the young group is stronger than that from old group (i.e., young > old). The figures in the **right** column show additional FCs revealed by the sliding windows and DAFA methods compared with the static method. Areas marked by circle indicated the additional altered FCs found by DAFA, compared with the sliding windows method.

As illustrated in Figure 4, a two-sample *t*-test was conducted on young and old groups to demonstrate the significant connections among all ROIs [*p* < 0.01, familywise error rate (FWE) corrected]. When comparing the results between the two groups, the FCs revealed by DAFA were similar to those from the static method. In detail, the connections within the DMN and the frontoparietal and cingulo-opercular networks were decreased, as well as connections between the frontoparietal and cingulo-opercular networks. Meanwhile, the connections within the sensorimotor network were increased, as well as the connections between the DMN and the other five networks and between the sensorimotor and frontoparietal/cingulo-opercular networks. In addition, both increased and decreased connections between the cingulo-opercular and cerebellum networks were found.

For the sliding window method, the group FC differences were similar to those of the static method, with the addition of altered connections between the cingulo-opercular and DMN and cerebellum networks. Further, compared with the static method, additional alterations in FC patterns (*p* < 0.01, FWE corrected) between the young and old groups were found with the DAFA method. In detail, decreased FCs were mainly within the cingulo-opercular networks, as well as in the connections between the cingulo-opercular and occipital/cerebellum networks. Increased FCs were mainly found in the connections between the DMN and the cingulo-opercular/occipital/cerebellum networks. Among these additional changed FCs, the main nodes were located in the postcingulate/precuneus/anterior cingulate cortex areas for the DMN, dorsolateral frontal cortex for the frontoparietal network, ventral prefrontal cortex/basal ganglia/mid-insula/thalamus for the cingulo-opercular network, the supplementary motor area for the sensorimotor network, the postoccipital region in the occipital network, and infcerebellum/medcerebellum for the cerebellum network (Figure 4). Noting that, compared with the sliding window method, additional altered FC patterns were found by DAFA. These altered FCs were: (1) decreased connections within cingulo-opercular network, (2) increased connections between DMN and cerebellum networks, and (3) decreased connections between cingulo-opercular and occipital networks.

### Relations Between Changed FCs and Behavioral Scores

**FIGURE 5:**
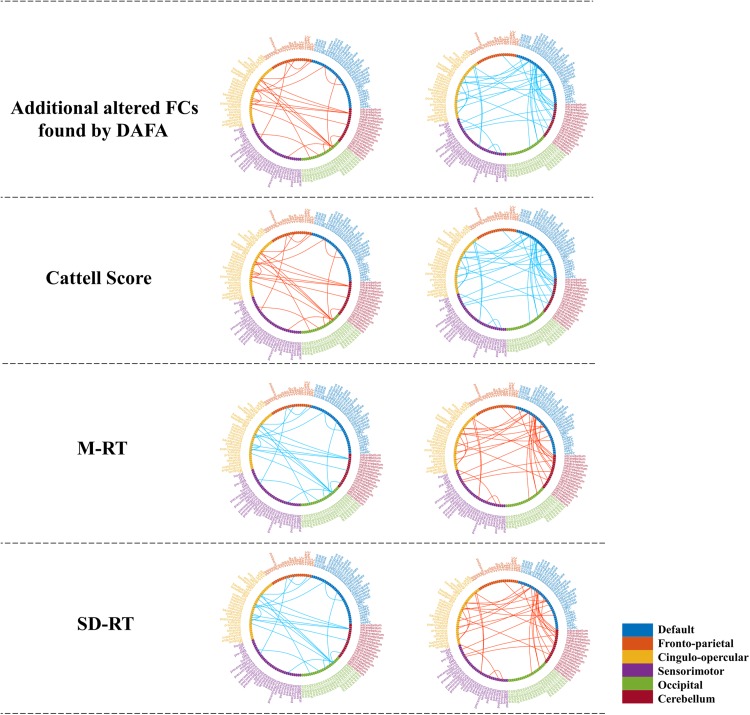
Significant relationships between the additional altered FCs (revealed by the DAFA method) and behavioral performance (*p* < 0.01). Figures on the top were additional FCs revealed by DAFA. Red lines indicate young > old; blue lines indicate old > young. The first column (from second row to the bottom row) shows the correlations between FCs (young > old) and the Cattell score/M-RT/SD-RT, while the second column (from second row to the bottom row) shows the correlations between FCs (young < old) and the Cattell score/M-RT/SD-RT.

To demonstrate the relationships between the altered FCs (revealed by the DAFA method) and behavioral performance, partial correlations between the altered FCs and behavioral scores (fluid intelligence and speed Choice Reaction Time) were calculated while controlling for gender, head motion and intracranial volume. Here, the Cattell score was related to fluid intelligence, while M-RT and SD-RT were related to the speed Choice Reaction Time. The detected FC patterns were significantly correlated with fluid intelligence and the speed Choice Reaction Time (*p* < 0.01). In detail, the FCs within the DMN and the frontoparietal, cingulo-opercular and cerebellum networks, the connections between the DMN and the frontoparietal/sensorimotor networks and the connections between the cingulo-opercular network and the occipital/cerebellum/frontoparietal networks were positively correlated with the Cattell scores. The FCs within the sensorimotor and occipital networks were negatively correlated with the Cattell scores, as well as the connections between the cerebellum network and the DMN and sensorimotor networks and the connections between the cingulo-opercular and sensorimotor networks. In addition, the relationships between the FCs and the M-RT/SD-RT scores had the opposite trend from that of the results of the Cattell scores. The details are shown in Figure 5.

## Discussion

In this paper, based on the hypothesis that most of the functional activities in young adults may reflect the “young FCs”, while those in old adults may largely reflect the “old FCs,” we proposed a method named DAFA to analyze the changes in FCs during the process of aging from a big data perspective. DAFA revealed additional significant, altered FC patterns between the young and old groups, which were ignored by the static method, and these changes were correlated with behavior scores (Cattell score, M-RT, and SD-RT), which might be indicative of cognitive decline during aging.

### DNN With AE Pretraining

For each training iteration, the sliding window method was used to calculate Pearson’s correlation coefficient for 160 ROIs from a subject’s brain, which enlarged the total sample size so that the autoencoder could examine the functional connectivity patterns more completely. The autoencoder is an unsupervised learning algorithm that can effectively mine hidden low-dimensional representations from data, which can then be used to reconstruct the original data (Tschannen et al., 2018). In this paper, we used a grid search to set and optimize the learning rate, batch size, epoch and other parameters of the autoencoder (Figure 2). The number of hidden nodes of AE is still open issue in deep learning. For example, if the number of the hidden nodes is based on the principle of minimum reconstruction error, the function of AE may present an identity transformation function and lose the ability of mining reasonable features. Therefore, referring to previous articles on the application of AE in fMRI (Suk et al., 2016; Guo et al., 2017) and empiricism setting of autoencoder, the hidden node of autoencoder in this work was set to 100. Finally, we obtained the model and corresponding parameters for the resting-state fMRI from CamCAN data. Unlike PCA or ICA, the autoencoder has no restrictions on whether the input data should be independent of each other and has no undetermined problem. It also revealed aging-related FCs from a comprehensive mining point of view with big data. The reconstruction error of 0.008 indicated that the extracted pattern was the representation of the FCs in low-dimensional space, which conveyed information for reconstructing the original signal to the greatest extent. The autoencoder could extract complex features; thus, the extracted features were combinations of linear and non-linear properties. The AE used in this work had a tradeoff between maximizing data representation and numerous restrictions (e.g., avoiding identity transformation and redundant feature extraction).

An AE-pretrained DNN can effectively reduce the training complexity and improve the training effect (Kim et al., 2016). In most cases, the DNN had been regarded as a black box. However, in deep learning-based fMRI studies, the weights between the input layer and the hidden layer of a neural network were interpreted as functional connectivity networks (Kim et al., 2016; Suk et al., 2016; Guo et al., 2017). In our case, these functional connectivity patterns can be interpreted as subnetworks of FNs during the resting-state or discriminable patterns comparing young and old groups. Noteworthy, there has been no fixed rule for hyper parameter settings in DNN models. Therefore, in our work, we used four layers for the network structure, and the number of nodes in each layer were set by experience for classification. This work was not concerned with the classification accuracy of the DNN model but instead was designed to see whether the relabeling results conform to our previous assumptions. Compared with SVM, the relabeling results obtained by the DNN rarely misclassified the FCs (e.g., the PCA + SVM results showed that more than 40% of the old subjects were relabel to the young group, as seen in Supplementary Material). Moreover, ICA + SVM requires that the samples be independent of each other, yet FCs from the same subject are not. Therefore, within the scope of this study, DNN was better than SVM. In addition, an unsupervised machine learning method, k-means clustering, was also applied to classify FCs in two classes based on Squalidean distance. And it had poor performance that the distribution of relabeled FCs by k-means clustering was scattered, and might be hard to detect underlying information of FCs (Supplementary Figure S3).

### Big Data Perspective

In the data preprocessing section, the Pearson’s correlation coefficient of time courses from every pair of ROIs was calculated by the sliding window method for one subject’s rs-fMRI. Several previous studies suggested that the window length should exceed the slowest frequencies (in this work, the time series of calculating FCs are high-pass filtered at 0.01 Hz) which commonly assumed to comprise the BOLD signal (Andrew and Michael, 2015; Leonardi and Van De Ville, 2015). Thus, the ideal window length was 100 s. Considering the TR of rs-fMRI in Cam-CAN dataset was 1.97 s, the window length of 50 time points was used in this work (i.e., 100/1.97≈50). This procedure enlarged the training dataset for the AE and satisfied the requirements of big data analysis processing flow (Cobb et al., 2018). From a big data perspective, with the increase in data volume, more credible results can be obtained, and errors caused by individual differences can be eliminated as much as possible (Xia and He, 2017; Peter and Jayati, 2018). However, a large data sample size almost inevitably leads to confusion effects, such as an incorrect attribution of samples (Smith and Nichols, 2018). In the proposed method, supervised learning was used to relabel all sliding window FCs. At the same time, deep learning performed better with large data samples (Lecun et al., 2015). Thus, combined with deep learning, DAFA enlarged the datasets and satisfied deep learning applications under big data circumstances, which led to more reliable results (Peter and Jayati, 2018). On the other hand, when the sample size increased, there was a subset in the sample that caused the statistical method to always produce significant statistical results and lead to incorrect conclusions (Smith and Nichols, 2018). Therefore, we averaged identical samples for each subject and took the resulting mean FC as that subject’s final FC, which made the samples used for statistical analysis independent and eliminated interference factors. The search-back step only considered the effect on the subject, not on the FCs derived from sliding window, which prevented confounding effects during the statistical analysis for big data (Smith and Nichols, 2018). Additionally, since we only analyzed young and old groups, each FC calculated from sliding window had a discriminatory prior (label), but according to the hypothesis, a small number of samples may have been mislabeled, so it was necessary to determine the samples corresponding to these false priors. Due to the poor performance of the clustering algorithm with high-dimensional data, it was not effective or sufficiently accurate to classify the samples; thus, in this article, the DNN was pretrained with the AE, and a supervised learning method was implemented to relabel and filter out the mixed samples.

### FC Changes in Aging

In our work, age-related FC changes were successfully revealed by the DAFA method. First, these changes included age-related decreased patterns, consisting of the FCs within the DMN and the frontoparietal and cingulo-opercular networks, as well as the connections between the frontoparietal and cingulo-opercular networks. Previous studies have suggested that decreased FC within the DMN and the cingulo-opercular network reflected the decline in cognitive function related to the attention, memory and executive functions in elderly individuals (Dijk et al., 2010; Ferreira and Busatto, 2013; Geerligs et al., 2015; Grady et al., 2016; Spreng et al., 2016), which is also supported by our findings on the correlation between behavioral score and the FCs in these networks. Regarding the decreased between-network FCs, the frontoparietal and cingulo-opercular networks have been hypothesized to support the top-down control of executive function (Dosenbach et al., 2008). The decreased FCs between the frontoparietal and cingulo-opercular networks might be caused by a reduction of harmonization of these networks and cognitive function loss in normal aging. Second, the connections within the sensorimotor network were increased, as were the connections between the sensorimotor and the frontoparietal/cingulo-opercular networks and between the DMN and the fronto-parietal/cingulo-opercular/sensorimotor/occipital/cerebellum networks. The sensorimotor function of elderly individuals shows a decline compared with young adults, and the increased FCs with the sensorimotor network might imply that a higher level of anticipated preparation was required for the decline of sensorimotor function (Mathys et al., 2014). Moreover, the increased connectivity between the sensorimotor and frontoparietal/cingulo-opercular networks might reflect a compensatory response to the dysfunction of other brain networks and neurotransmitter decline (He et al., 2016). Furthermore, the increased FCs between the DMN and the other FNs have also been found by previous studies (Zhang et al., 2015; Grady et al., 2016; Spreng et al., 2016; Damoiseaux, 2017), which might be due to the DMN playing an enhanced intermediary role in regulating primary and higher-order networks (Margulies et al., 2016; Kernbach et al., 2018). Third, both increased and decreased connections between the cingulo-opercular and cerebellum networks were also found, which might reflect other dysfunction in elderly individuals. In brief, the results from DAFA were consistent with the abovementioned studies to some extent.

Our DAFA method yielded additional changed FCs compared to the static method, which showed decreased FCs compared with the young group were mainly in the connections between the cingulo-opercular and occipital/cerebellum networks. Previous studies have implied that the cingulo-opercular network is important for set maintenance and other functions, including processing negative effects, pain and cognitive control (Dosenbach et al., 2008; Church et al., 2009; Sylvester et al., 2012); additionally, an alertness study found that the cingulo-opercular network showed a task-positive response in an event-related design with auditory and visual stimuli (Coste and Kleinschmidt, 2016). Therefore, the decreased FCs between the cingulo-opercular and occipital networks in our work likely implied the decline in high-order functions (e.g., set-maintenance and alertness functions). Furthermore, the cerebellum is associated with many cognitive functions involving emotion, executive function, language and working memory (Keren-Happuch et al., 2014). A decreased FC between the cingulo-opercular and cerebellum networks might suggest a loss in the harmonization of these networks in elderly individuals. Meanwhile, the increased additional FCs were mainly in the connections between the DMN and cingulo-opercular/occipital/cerebellum networks, which was consistent with the trend in the static results, thereby adding more detailed evidence to the FC changes in aging. In addition, the result of additional FCs detected via DAFA might imply that two kinds of neural circuits (decreased connections between the cingulo-opercular network and the occipital/cerebellum networks, and increased connections between DMN and cingulo-opercular/occipital/cerebellum networks) existed, which perhaps were corresponding to harmonization loss and compensation mechanism, respectively.

### Limitations

The limitations of current work are following: first, the aim of this work was to detect the information between health young group and old group (aging), and the current age division was based on the published paper of Cam-CAN open dataset (Shafto et al., 2017). However, a finer bin-based age classification may be investigated in the future effort. Second, considering deep learning performed better with large data samples (Lecun et al., 2015) and it perhaps has satisfied performance on mixed labels (Guan et al., 2017), we proposed a new method combining deep learning with neuroimaging big data, to fully explore the potentials of data with new hypotheses, and some replicate existing findings were also obtained. However, our method may need to be further improved, e.g., heuristic/clinically inspired method could be developed to prevent the autoencoder on digesting irrelevant connections. Third, the deep learning models applied in this study were an autoencoder and a DNN. During the training process, these models required initialization of their hyper parameters, which generally relied on user experiences. For example, there is no gold standard for the number of hidden nodes in an auto-encoder. Another is that performing the proposed DAFA method requires GPUs to train the deep learning models; otherwise, it would consume more time than traditional methods such as ICA and SVM. The current version of DAFA codes was available on https://github.com/Xin-cqu/DAFA. At last, more efforts were needed to verify that DAFA might be a promising method for exploring important information in other fMRI studies.

## Conclusion

In this work, we developed a new method named DAFA to deeply detect important information about age-related FC changes. The results demonstrated that DAFA had the following advantages: (1) the potential confused relationships in the data was taken into account and combined with deep learning methods, DAFA could detect the complex information of functional connectivity networks for normal aging in a more comprehensive way; (2) from big data perspective, DAFA has enlarged the dataset and reduced the cofounds effect of statistical analysis resulting in improving the reliability of the analysis; and (3) DAFA was constructed as a new method to detect underlying important brain information from fMRI analysis. Additionally, it may be a promising method for exploring important alteration information in fMRI studies on diseases such as Alzheimer’s disease and schizophrenia.

## Data Availability Statement

Publicly available datasets were analyzed in this study. This data can be found here: https://camcan-archive.mrc-cbu.cam.ac.uk/dataaccess/.

## Ethics Statement

The studies involving human participants were reviewed and approved by the Cambridgeshire 2 (now East of England – Cambridge Central) Research Ethics Committee. The patients/participants provided their written informed consent to participate in this study.

## Author Contributions

LD and JC conceived the project. XW and LD implemented the code of the method and wrote the manuscript. XL and JY preprocessed the MRI data. LD and CL supervised the analysis of FC patterns discovered by the method. HL, JX, DY, and CL provided critical suggestions for the manuscript.

## Conflict of Interest

The authors declare that the research was conducted in the absence of any commercial or financial relationships that could be construed as a potential conflict of interest.

## Footnotes

1 http://www.cam-can.com

2 http://www.fil.ion.ucl.ac.uk/spm
