# Supplementary material for: Detecting the Information of Functional Connectivity Networks in Normal Aging Using Deep Learning From a Big Data Perspective
Source: Front Neurosci. 2020 Jan 17;13:1435. doi: 10.3389/fnins.2019.01435 (PMC6978665; doi:10.3389/fnins.2019.01435)
Supplement: Supplementary file 1 [file Table_1.DOCX]

Supplementary Material

To illustrate the advantages of DAFA in exploring the changes of brain functional connectivity in the aging population, we compared PCA + SVM and ICA + SVM methods. Among them, PCA and ICA have 20 dimensionality reduction components. SVM uses libsvm toolkit (https://www.csie.ntu.edu.tw/~cjlin/libsvm/) and default parameters. In PCA+SVM case, considerable 97.53% of FCs in young group were young FC, and exceptionally high 46.31% of FCs in old group were relabeled as young; such percentage both decreased in the case of ICA+SVM which were 82.1% and 25.2%, respectively (Table S1). Recalling our assumption most of the functional activities via FCs at different times in young adults may reflect the “young FCs” (FCs which reflect intrinsic brain activities in young adults), while those in old adults may largely reflect the “old FCs” (FCs which reflect intrinsic brain activities in old adults)., in PCA+SVM case, 97.5% of FCs in young group were considered as “young FCs” which made little difference with the results via sliding window method, while 46.3% of FCs in old group were considered as “young FCs” which lead to a large variance of “young FCs” percentage. Such limitations would be overcome by ICA+SVM method, however ICA requires input data have an independent relationship each other which were contrary to our situation. At last, the result of DAFA matched our hypothesis better.


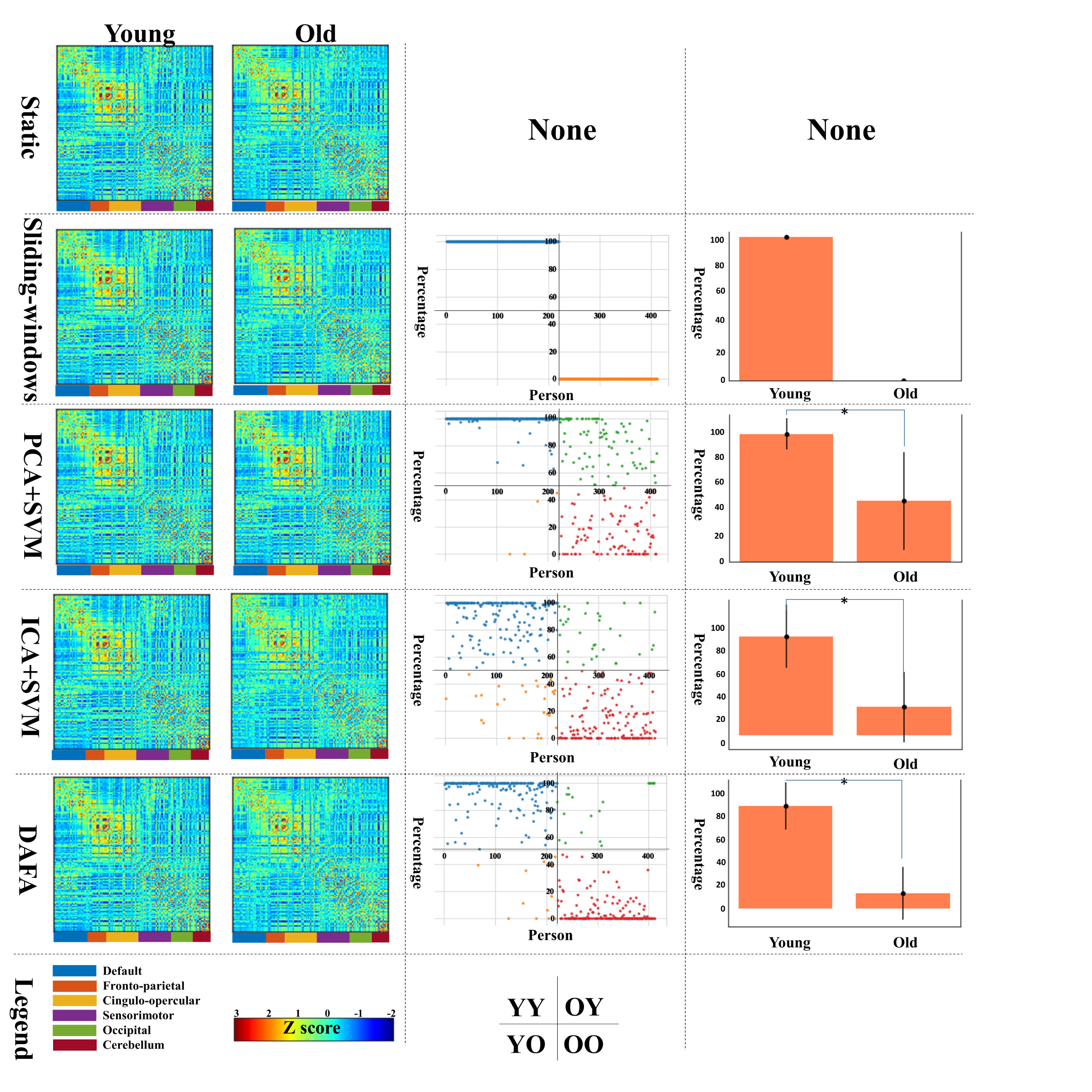


**Figure S1.** The first column shows the average FC patterns of the young group and old group on the left and right, respectively; the second column shows the distribution of “young FCs” (samples in blue indicate individuals in the young group whose percentage of “young FCs” is greater than 50%; samples in orange indicate individuals in the young group whose percentage of “young FCs” is equal to or below 50%; samples in green indicate individuals in the old group whose percentage of “young FCs” is above 50%; and samples in yellow indicate individuals in the old group whose percentage of “young FCs” is equal to or less than 50%); the third column shows the mean and standard deviation of “young FCs” in the two groups. YY indicates samples in the young group for whom more than 50% of the FCs were relabeled to young; YO indicates samples in the young group for whom more than 50% of the FCs were relabeled to old; OO indicates samples in the old group for whom more than 50% of the FCs were relabeled to old; and OY indicates samples in the old group for whom more than 50% of the FCs were relabeled to young.

**Table S1: the percentage of “young FCs” for the five folds CV**

| Methods | Group | Fold 1 | Fold 2 | Fold 3 | Fold 4 | Fold 5 | Average |
| --- | --- | --- | --- | --- | --- | --- | --- |
| PCA+SVM | Young | 91.8 | 98.9 | 97.7 | 100 | 99.3 | 97.5±2.9 |
|  | Old | 41.4 | 42.8 | 47.8 | 48.2 | 50.9 | 46.3±3.9 |
| ICA+SVM | Young | 75.3 | 82.3 | 83 | 85.5 | 82 | 82.1±3.8 |
|  | Old | 25.4 | 28.9 | 19.5 | 26.6 | 24.3 | 25.2±3.5 |
| DAFA | Young | 83.6 | 91.3 | 89.1 | 94.5 | 90.7 | **89.8±4.0** |
|  | Old | 10.6 | 16.9 | 15.4 | 15.1 | 7.6 | **13.2±3.8** |

We proposed the hypothesis that most of the functional activities in young adults may reflect the “young FCs” (FCs which reflect intrinsic brain activities in young adults), while those in old adults may largely reflect the “old FCs” (FCs which reflect intrinsic brain activities in old adults). To demonstrate above assumption, we plotted the distributions of principal FCs (principal components) in young and old groups. Figure S2 showed that some FCs with young or old labels via sliding window method were mixed. However, noting that for FCs of one subject (especially for altered principal FCs, see Figure S2), most of labels were able to reflect the “young FCs” and “old FCs”, respectively. And the remaining information of eight PCs can be seen in Figure S4. In addition, here, we also used an unsupervised machine learning method, k-means clustering, to classify FCs in two classes based on Squalidean distance. For young group, 66.7% of FCs were clustered as “young FCs”, while 67.0% of FCs were clustered as “old FCs” for old group. However, the distribution of relabeled FCs by k-means clustering was scattered, and might be hard to detect underlying information of FCs (Figure S3).


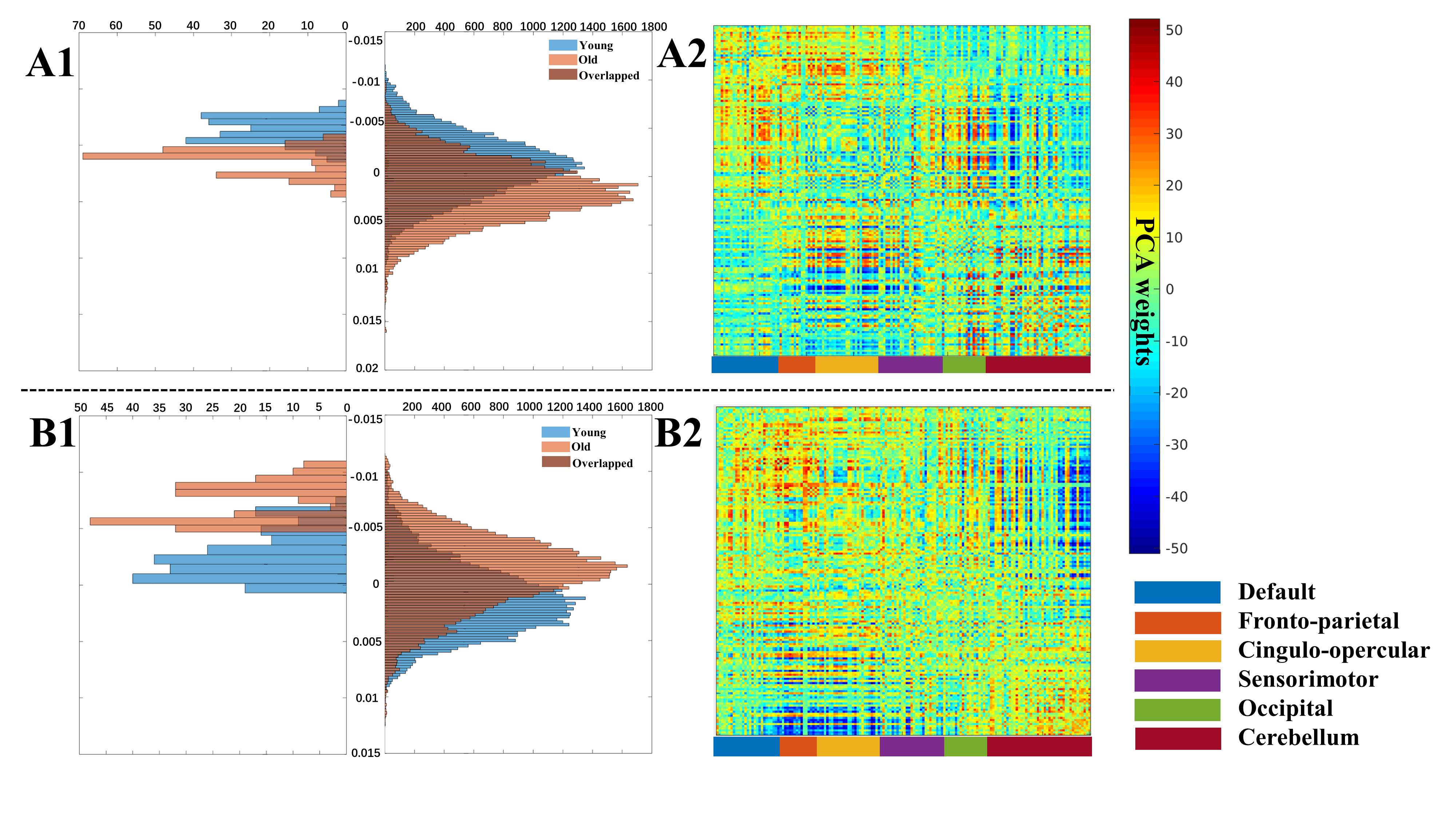


**Figure S2** The distribution of two example principal components (A is third PC and B is fourth PC, which were altered PCs between young and old groups) with young and old labels. In figure A1 and B1, the y axis is the PCA value, the x axis is the count. The left part of A1 and B1 are the distribution of a young subject and an old subject in the PCs, respectively. The right part of A1 and B1 are the distribution of all young and old subjects in the PCs, respectively. The light brown indicates the young group, the blue indicates old group and the dark brown indicates the overlap part of young and old groups. A2 and B2 are the visualization of corresponding PCs (i.e. network patterns).


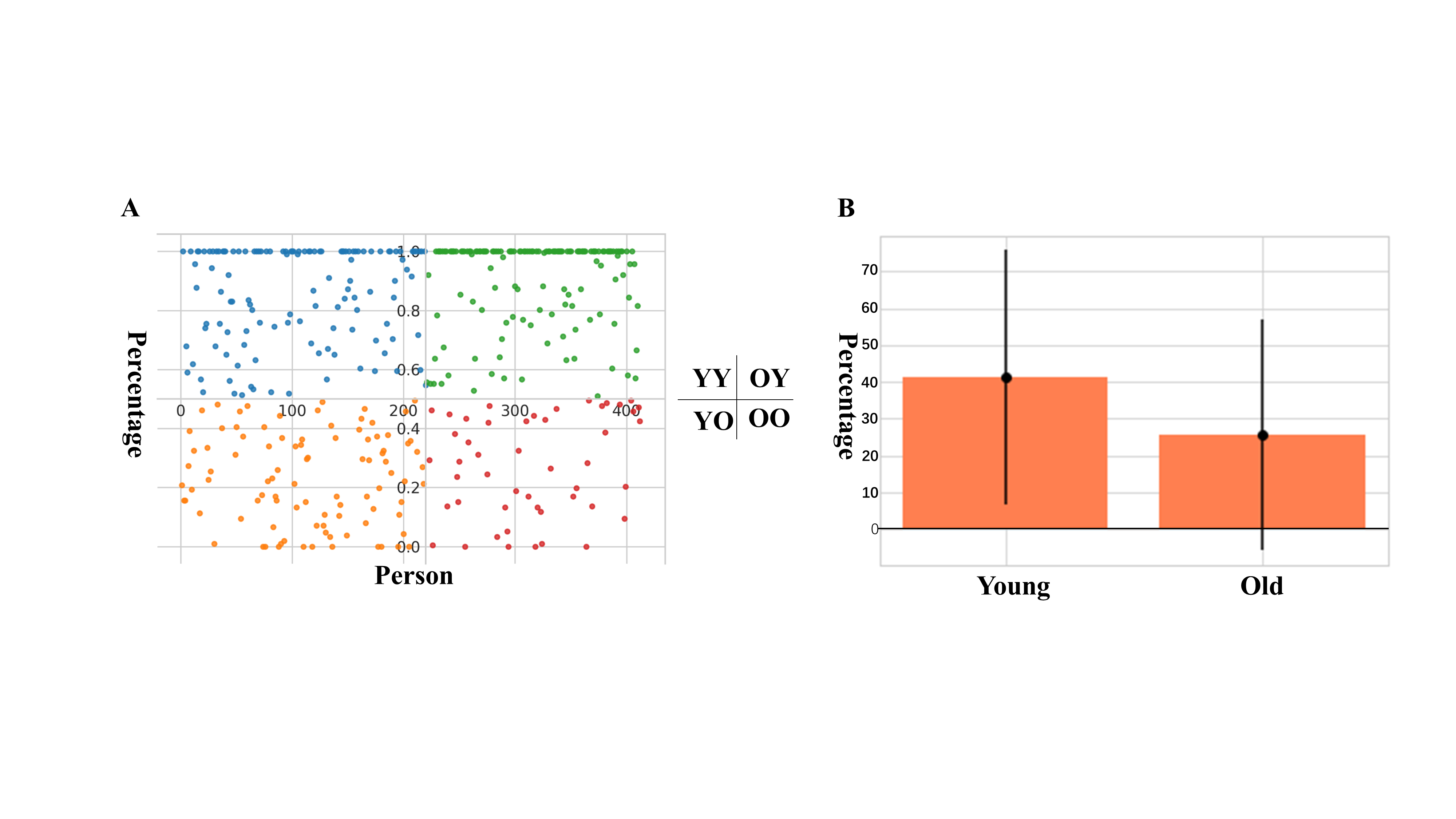


**Figure S3** A. the “young FCs” distribution of K-means cluster method for classify the “young FCs” and “old FCs”. X axis is the samples of 412 subjects, Y axis is the percentage of “young FCs” in each subject. B. the mean and standard deviation of “young FCs” in the two groups (samples in blue indicate individuals in the young group whose percentage of “young FCs” is greater than 50%; samples in orange indicate individuals in the young group whose percentage of “young FCs” is equal to or below 50%; samples in green indicate individuals in the old group whose percentage of “young FCs” is above 50%; and samples in yellow indicate individuals in the old group whose percentage of “young FCs” is equal to or less than 50%); YY indicates samples in the young group for whom more than 50% of the FCs were relabeled to young; YO indicates samples in the young group for whom more than 50% of the FCs were relabeled to old; OO indicates samples in the old group for whom more than 50% of the FCs were relabeled to old; and OY indicates samples in the old group for whom more than 50% of the FCs were relabeled to young.


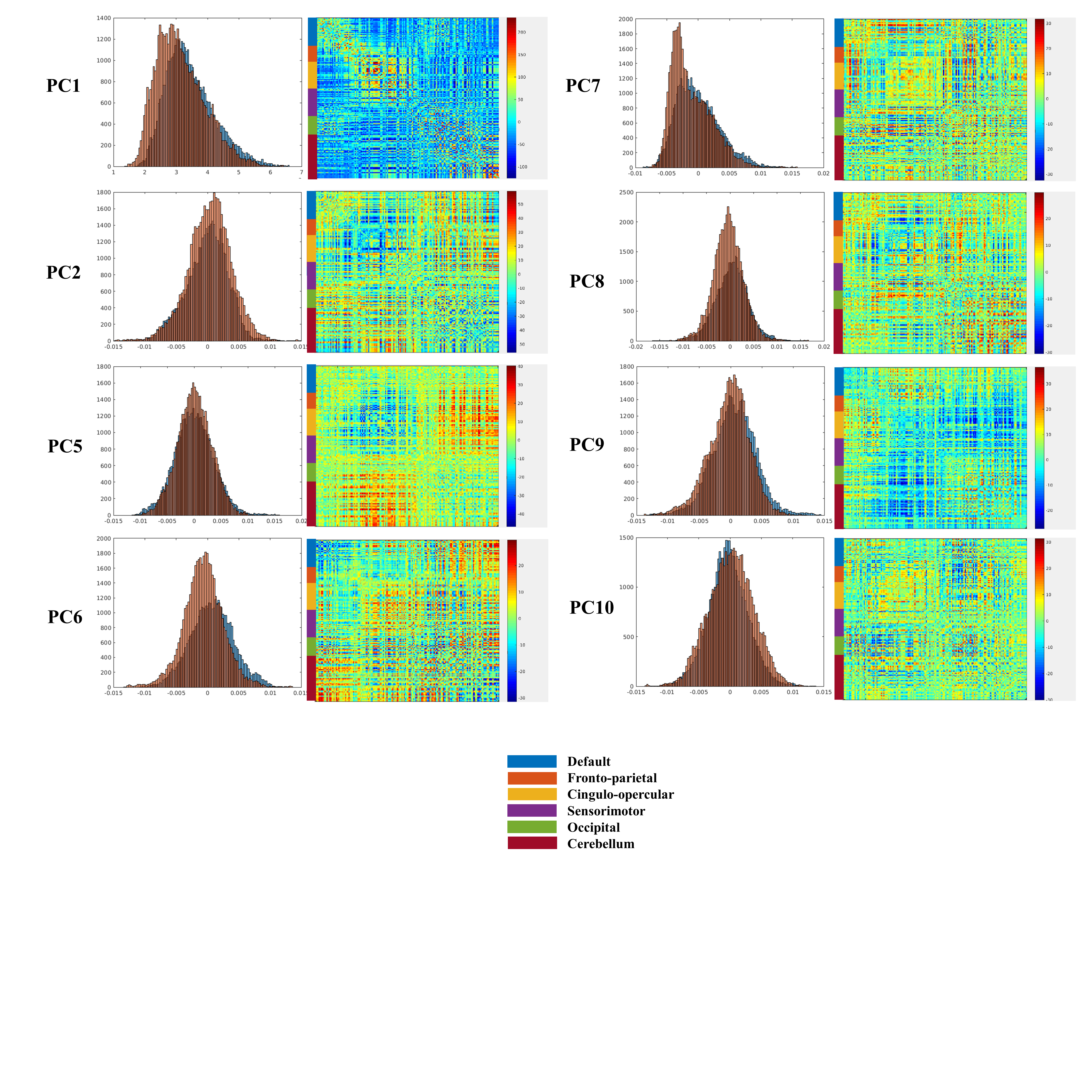


**Figure S4** The left part in each subfigure was the distribution of eight PCA components of first ten comparing between young and old group. The x axis was the PCA value, the y axis was the sum of occurrence of specific PCA value. And the right part in each subfigure was the corresponding visualization of PCA components’ network pattern. The light brown indicating that value in young group, the blue indicating that value in old group and the dark brown indicating the overlap part of both young and old groups.

**References**

Biswal, B.B., Mennes, M., Zuo, X., Gohel, S., Kelly, C., Smith, S.M., et al. (2010). Toward discovery science of human brain function. *Proceedings of the National Academy of Sciences of the United States of America* 107(10)**,** 4734-4739.

Biswal, B.B., Yetkin, F.Z., Haughton, V.M., and Hyde, J.S. (1995). Functional connectivity in the motor cortex of resting human brain using echo‐planar mri. *Magnetic Resonance in Medicine* 34(4)**,** 537-541. doi: 10.1002/mrm.1910340409.

Karahanoglu, F.I., and Van De Ville, D. (2015). Transient brain activity disentangles fMRI resting-state dynamics in terms of spatially and temporally overlapping networks. *Nat Commun* 6**,** 7751. doi: 10.1038/ncomms8751.

Liu, X., and Duyn, J.H. (2013). Time-varying functional network information extracted from brief instances of spontaneous brain activity. *Proc Natl Acad Sci U S A* 110(11)**,** 4392-4397. doi: 10.1073/pnas.1216856110.
